# Supplementary material for: Comprehensive Annotation and Functional Exploration of MicroRNAs in Lettuce
Source: Front Plant Sci. 2021 Dec 24;12:781836. doi: 10.3389/fpls.2021.781836 (PMC8739914; doi:10.3389/fpls.2021.781836)
Supplement: Supplementary file 2 [file Data_Sheet_8.ZIP › GSM2230748.plot/Lsa-miR396d_Lsat_1_v5_gn_8_127960.1_2426_TPlot.pdf]

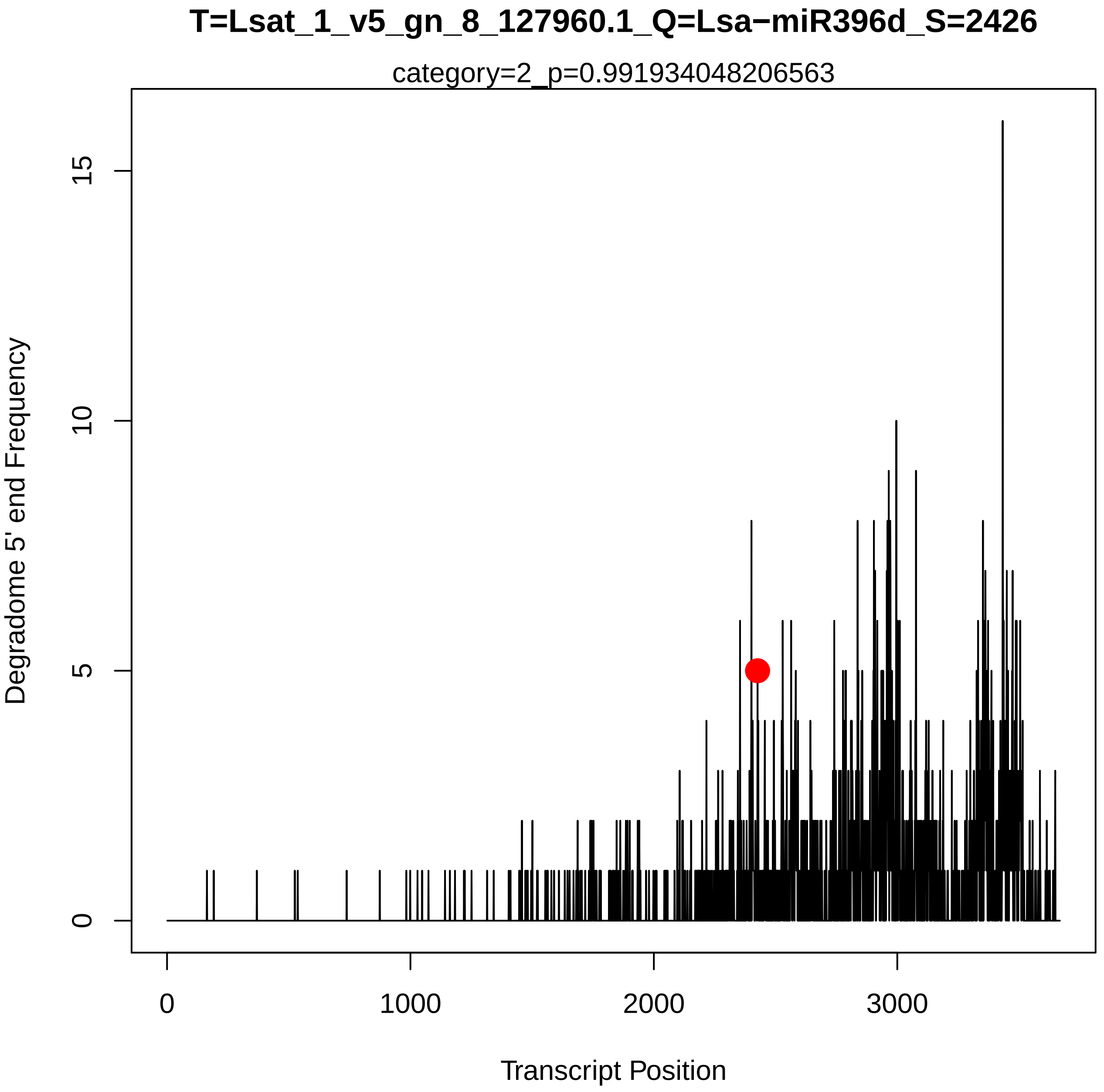

T=Lsat_1_v5_gn_8_127960.1_Q=Lsa−miR396d_S=2426
category=2_p=0.991934048206563
Degradome 5' end Frequency
0
5
10
15
0
1000
2000
3000
Transcript Position
